# Multiple Roles of Photosynthetic and Sunscreen Pigments in Cyanobacteria Focusing on the Oxidative Stress

**DOI:** 10.3390/metabo3020463

**Published:** 2013-05-30

**Authors:** Naoki Wada, Toshio Sakamoto, Seiichi Matsugo

**Affiliations:** School of Natural System, College of Science and Engineering, Kanazawa University, Kakuma, Kanazawa 920-1192, Japan; E-Mails: naoki-wada@se.kanazawa-u.ac.jp (N.W.); s-matsugoh@se.kanazawa-u.ac.jp (S.M.)

**Keywords:** anhydrobiosis, antioxidant, glycosylation, oxidative stress, photosynthetic pigment, sunscreen pigment, UV stress

## Abstract

Cyanobacteria have two types of sunscreen pigments, scytonemin and mycosporine-like amino acids (MAAs). These secondary metabolites are thought to play multiple roles against several environmental stresses such as UV radiation and desiccation. Not only the large molar absorption coefficients of these sunscreen pigments, but also their antioxidative properties may be necessary for the protection of biological molecules against the oxidative damages induced by UV radiation. The antioxidant activity and vitrification property of these pigments are thought to be requisite for the desiccation and rehydration processes in anhydrobiotes. In this review, the multiple roles of photosynthetic pigments and sunscreen pigments on stress resistance, especially from the viewpoint of their structures, biosynthetic pathway, and *in vitro* studies of their antioxidant activity, will be discussed.

## 1. Introduction

### 1.1. Metabolites in Cyanobacteria

Cyanobacteria are the first prokaryote that appeared on the earth to acquire the oxygenic photosynthetic ability. Cyanobacteria occupy diverse ecological niches, adapting to various extreme environments, such as high or low temperatures, highly acidic or basic pH, high salt concentrations, desiccation, and are now widely distributed on the Earth [1]. Not only did aquatic ones adapt to fresh-water and marine environments, but terrestrial ones are also known. Various metabolites are produced in cyanobacteria, as diverse as their habitats, for example, cyanotoxin, siderophores, phytohormones, grazer deterrents, antiviral compounds, anticancer compounds, antimicrobial compounds, antifungal compounds, antiplasmodial compounds, immunosuppressive compounds, algicides, protease inhibitors, photoprotective compounds, and antibiotic compounds. These metabolites can be classified based on their structures, such as polyketides, amides, alkaloids, fatty acids, indoles, and lipopeptides. These are useful bioactive substances, and it is obvious that cyanobacteria are important biological resources. Many articles have been published about the structures and functions of the bioactive substances, as well as their industrial applications [2,3,4].

### 1.2. Anhydrobiosis of Terrestrial Cyanobacteria

Certain desiccated organisms have no metabolic activity and are able to rapidly resume metabolism upon rehydration in a phenomenon termed “anhydrobiosis”. It is believed that protein denaturation while in a desiccated state is prevented by their own metabolites. However, the detailed mechanism still remains unclear. Some microorganisms contain a great deal of extracellular polysaccharide (EPS) in a structure directly attached to the cell wall and/or physically interacting with other cells. In cyanobacteria the production of EPS is widely known and EPS plays a major role in protecting cells from various stresses in harsh habitats, including extreme desiccation. The desiccation tolerance in *Nostoc commune* is strongly associated with EPS because removal of EPS causes significant damage to cells during desiccation [5].

Nonreducing disaccharides, such as trehalose and sucrose, are believed to have an important role in one of the proposed anhydrobiosis mechanisms [6]. Among anhydrobiotic gram-negative bacteria, *Escherichia coli* and *Pseudomonas putida* are known to accumulate special metabolites, such as trehalose and hydroxyectoin, in their cells. The anhydrobiotic cyanobacterium *Nostoc commune* in a desiccated state accumulates trehalose intracellularly [7]. The role of nonreducing disaccharides can be divided into two categories. One anhydrobiosis mechanism is due to vitrification by integrating nonreducing disaccharides into a vitreous cytoplasmatic matrix [8]. The other anhydrobiosis mechanism is due to the substitution of water molecules, which are lost through desiccation, with disaccharide molecules [6]. Higher-order protein structure can be maintained by stabilizing hydrogen bonds in a desiccated state; thus, the loss of protein function can be prevented [9]. It has also been reported by numerous groups that trehalose contributes to anhydrobiosis more than sucrose [10]. The glass-transition temperature (Tg) of trehalose is 110 °C, which is much higher than that of sucrose (65 °C). Thus, trehalose can maintain glassy state even at high temperatures, which results in a better ability to maintain a matrix structure when compared to sucrose. However, some reports indicate that trehalose alone cannot promote anhydrobiosis [11]. The aquatic cyanobacterium *Nostoc verrucosum* produces massive extracellular polysaccharides and accumulates trehalose in response to desiccation, although *Nostoc verrucosum* cells are sensitive to desiccation, unlike the anhydrobiotic cyanobacterium *Nostoc commune* [12].

Based on the above contradiction, it is necessary to consider the contribution from other substances. It is believed that anhydrobiosis in cyanobacteria involves various substances, such as saccharides (including polysaccharides, oligosaccharides, and disaccharides) and proteins (including peptides), which complement each other. As one example, it was suggested that water stress protein is immediately synthesized during desiccation and contributes to maintain the matrix structure [13]. In recent years, it has been found that heat shock proteins (HSP), which are synthesized in response to heat stress, are also effective against desiccation [13,14]. Late embryogenesis abundant (LEA) proteins were initially discovered in cottonseeds and classified into at least four groups, based on their amino acid sequences. They are well known to accumulate during desiccation. Recently, Shimizu *et al.* found that group 3 LEA proteins play a role in reinforcing the glassy matrix of trehalose by changing its geometry from random coils to α-helical coiled coils [15]. As far as we know, the genes encoding LEA protein analogs have not yet been found in cyanobacteria. An extracellular matrix protein, water stress protein (WspA), is abundantly produced in EPS producing cyanobacteria *Nostoc commune* and *Nostoc verrucosum* [12], but there is no apparent structural similarity between WspA and LEA proteins. The WspA protein may have a protective role for maintaining cells in a desiccated state, however, it remains to be demonstrated in future studies.

### 1.3. Reactive Oxygen Species (ROS) Induced Damages and Stress Responses

Aerobes obtain energy through the respiratory chain by reducing oxygen. Reactive oxygen species (ROS), such as superoxide anion radical (O_2_^−^•), hydrogen peroxide (H_2_O_2_), and hydroxyl radical (OH•), which are produced as inevitable by-products during respiration, damage DNA, proteins, and membrane lipids by oxidization. In O_2_-producing photosynthetic organisms, including cyanobacteria and algae, ROS can also be produced during photochemical reactions and the photosynthetic electron transport [16]. In addition, high light illumination increases ROS production due to an excessive amount of energy to photochemical reactions. Photosynthetic organisms show various responses in order to avoid ROS over-production and the damages on biomolecules induced by ROS. They protect themselves by the following mechanisms, shading light through sunscreen pigments, controlling photosynthetic electron transport by favoring photosystem I or photosystem II, and activation of the system quenching the ROS produced (antioxidant system) [16]. Substances that diminish ROS activities are called antioxidants and enzymes that play similar roles are called antioxidant enzymes. A balance between oxidation by ROS and reduction by antioxidants and antioxidant enzymes is usually maintained (redox balance). However, redox balance can be greatly disrupted by a loss in antioxidant activity due to environmental stress conditions, including desiccation. Once controls of the redox balance are disrupted irreversibly, homeostasis of cells can be collapsed and result in cell death.

DNA is a relatively stable molecule, though the purine and pyrimidine bases can be oxidized by strong ROS, such as hydroxyl radical [17]. Hydroxyl radical is produced from hydrogen peroxide by catalysis of Fe^2+^ (Fenton’s reaction). Oxidation of DNA causes a loss in homeostasis because it affects both the replication and translation processes. In proteins, amino acid residues such as proline, arginine, lysine, and threonine can be carbonylated by oxidation. Carbonylated proteins are not only physiologically inactive but are also easily hydrolyzed by enzymes to be removed. It is known that carbonylated proteins are accumulated in desiccation intolerant cells during drying because desiccation causes strong oxidative stress and protein carbonylation exceeds the removal of modified proteins [18]. Biomembranes are normally in contact with many water molecules and maintain their liquid crystalline state, which is important to keep biomembrane functions. Membrane fluidity and its selective permeability can be lost by the oxidation of unsaturated lipid molecules in the cell membrane by ROS via excess peroxidation and de-esterification under desiccated conditions. Moreover, packing of the polar head groups of membrane lipids becomes dense, which causes the packing of alkyl chains also to be dense [19]. Under such stressed conditions, the phase transition temperature (Tm) of membrane lipids increases so that the liquid crystalline state becomes a gel state, even at room temperature. As a result, biomembranes are irreversibly damaged and organisms die. Thus, anhydrobiotes are thought to be equipped with mechanisms to lower the phase transition temperature in membrane lipids to adapt to extreme desiccated conditions. As increasing the amount of unsaturated lipids lowers the Tm value, the fluidity of membrane lipids increases but the resistance against ROS decreases because polyunsaturated fatty acids are highly sensitive to oxidation; obviously the controls of membrane lipid unsaturation are a trade-off phenomenon to avoid the damaging of biomembranes under stressed conditions. Thus, in both desiccation and rehydration processes, complex mechanisms against ROS-induced damages are required and the cellular responses should be controlled in a sophisticated manner. Desiccation tolerance is a common phenomenon in plants; therefore membrane behavior during desiccation has been extensively studied [20]. Further research in the control of membrane lipid unsaturation is necessary to generalize this idea into anhydrobiosis to protect biomembranes from oxidative damage, which is always associated with environmental stresses.

In this review, the antioxidant properties of the photosynthetic pigments, chlorophylls and carotenoids, and the sunscreen pigments, mycosporine-like amino acids (MAAs) and scytonemin, which are bioactive substances in cyanobacteria, are summarized. In particular, we focus on their glycosylated derivatives and protein-bound forms, which are thought to be relevant to molecular mechanisms of anhydrobiosis. The complete explication of the multi-functionality of these pigments *in vivo* is desired in future. However, cyanobacteria are expected to be, as at least one of, the great sources of novel metabolites useful for human health because all antioxidative pigments listed above are produced in cyanobacteria including ones adapted to extreme environments.

## 2. Photosynthetic Pigments

### 2.1. Chlorophylls and Their Derivatives

Chlorophyll is a ubiquitous photosynthetic pigment and functions in photosystems, as well as light-harvesting antenna. Known chlorophylls are classified into seven isoforms, *a* through *f*, depending on the difference in ring substituents. Chlorophyll *e* is not used as a technical term because it is considered as artificial product. The predominant isoform commonly found in cyanobacteria is chlorophyll *a*. In recent years, it has been found that *Acaryochloris marina*, a unique cyanobacterium, contains isoforms *d* and *f* [21]. Chlorophylls have a characteristic absorption spectrum with the red and blue absorption bands in visible light region due to the wide π-conjugated plane [22]. For example, chlorophyll *a* has two absorption bands at 660 nm and 430 nm in diethyl ether. The blue absorption band is commonly called the Soret band. These bands further split into two more bands, respectively, because of their asymmetrically substituted side groups. The absorbance variety of chlorophylls allows the adaptation to specific light regimes in their habitats.

Cyanobacteria form a unique light-harvesting complex (phycobilisome), which is regularly associated with photosystem II to serve light energy transfer. It consists mainly of two kinds of phycobiliproteins; phycocyanin as a rod, and allophycocyanin as a core. In some cyanobacteria, it consists of phycoerythrin as an additional rod protein. The size and protein composition of this complex changes in response to the light and nutrition regimes. The amount of one or both of phycoerythrin and phycocyanin can change through a chromatic adaptation mechanism [23]. In low intensity light, synthesis of phycoerythrin is promoted to elongate rod structures. It has been reported that phycoerythrin is sensitive to pH, salt concentration, temperature change, desiccation, and light stress [24]. Phycobilins are chromophores covalently binding to phycobiliproteins. The phycobilins are classified into phycocyanobilin, phycoerythrobilin, phycoviolobilin, and phycourobilin according to their structures. These phycobilins absorb their specific wavelengths of light between 470 and 650 nm, which are shorter wavelength regions than red absorption band (Q-band) around 660 nm of chlorophylls. The higher excitation energy makes it possible for phycobilins to transfer photo-energy to chlorophylls.

### 2.2. Antioxidant and Pro-Oxidant Activity of Chlorophylls and Phycobilins

Although the antioxidant properties of chlorophylls have also been elucidated, *in vitro* studies show that various chlorophyll derivatives react with the 1,1-diphenyl-2-picrylhydrazyl radical (DPPH), one of the stable organic radicals. It was discovered in experiments using DPPH and 2,2'-azinobis(3-ethylbenzothiazoline-6-sulfonic acid) (ABTS) radicals, that radical scavenging activity by chlorophyll *a* is far stronger than that of chlorophyll *b* [25]. In addition to this, metal-free chlorophyll derivatives, which lose metal ions in center, such as chlorins, pheophytins, and pyropheophytins, have significantly lower radical scavenging activity compared with that of authentic chlorophylls containing metal ions in their centers, such as Mg-chlorophyll, Zn-pheophytins, Zn-pyropheophytins, Cu-pheophytins, and Cu-chlorophyllins. Contrary to this, chlorophylls are widely known as sensitizer for the production of singlet oxygen, that means chlorophylls function as a pro-oxidant [26]. It is still difficult to conclude if chlorophylls really function as an antioxidant *in vivo*.

Phycobilins have a linear tetrapyrrole structure, while chlorophylls contain the central metal ion with a closed ring structure. This characteristic structure of phycobilins is in common to that of biliverdin and bilirubin, which are generated by opening the heme ring. Biliverdin and bilirubin have strong antioxidant properties, so that phycobilins are also expected to have the similar antioxidant activity. Phycocyanobilin, isolated from *Spirulina platensis*, is known to prevent peroxidation of linoleic acid [27]. According to the *in vitro* experiments regarding lipid peroxidation using phosphatidylcholine liposomes, scavenging ROS by extracellularly scattered phycocyanobilin, inhibited the radical initiation for peroxidation of unsaturated lipid molecules. However, it is also indicated that phycobiliprotein can potentially function as a pro-oxidant. Phycocyanin certainly exhibits radical scavenging activity in dark environments, though it generates hydroxyl radicals in light environments. It is reported that the denaturation of phycocyanin proteins by surfactants, urea, and alkaline can completely diminish hydroxyl radical production while radical scavenging activity still remains [28]. Therefore, it is possible that phycobilin, itself, functions as an antioxidant while protein-bound phycobilin functions as a pro-oxidant.

### 2.3. Carotenoids and Their Derivatives

Common structural character of carotenoids is long π-conjugated polyene structure with several methyl substituents, which is the unique chromophore of carotenoids. Carotenoids commonly have three absorption bands in the region between 400 and 500 nm. They are usually strongly hydrophobic due to the long unsaturated alkene chain, so that they localize and function in biomembranes. Many kinds of carotenoids have cyclohexene end groups at the one side or both sides. The major carotenoids in cyanobacteria are β-carotene, and zeaxanthin and nostoxanthin as hydroxy-derivatives, and echinenone and canthaxanthin as keto-derivatives. Myxoxanthophyll is a major carotenoid glycoside having unique linkage, in which glycoside links to a hydroxyl group at C-2' of the Ψ end group of the carotenoid in (2'S)-configuration. This structure has been found only in cyanobacteria. Several myxoxanthophyll derivatives, α-L-rhamnoside, α-L-fucoside, α-L-chinovoside, and 2,4-di-O-methyl-α-L-fucoside, have also been identified in cyanobacteria [29]. Orange carotenoid protein (OCP), a water-soluble 35 kDa protein, was identified in three genera of cyanobacteria. Crystal structure analysis of OCP in *Arthrospira maxima* revealed that the carotenoid, 3'-hydroxyechnenone, binds to the protein non-covalently between N- and C-terminal domains [30]. The action spectrum of the phycobilisome fluorescence quenching exactly matches the absorption spectrum of the carotenoid, 3'-hydroxyechinenone in OCP. The light absorption by OCP induced structural changes in the carotenoid and protein, which results in the conversion from a stable orange form into a red, relatively unstable, active form. The red form accumulated under the condition in which photo-protection is required [31,32]. These substituted carotenoids might respond to various kinds of stresses, the same as carotenoids, though the relationship with anhydrobiosis is still unknown.

### 2.4. Antioxidant Activity of Carotenoids

Carotenoids are not directly involved in photochemical reactions, so their main function is thought to protect photosynthetic machinery from oxidative damage by acting as sunscreen pigments and antioxidants. The amounts of echinenone and myxoxanthophyll increase about 1.5 times after exposure to UV-B light, in terrestrial cyanobacterium *Nostoc commune* [33]. The amounts of carotenoids are also increased slightly by UV-A exposure in aquatic cyanobacterium *Oscillatoria* sp. [34]. These results suggest both carotenoids and their glycosides function as photo-protectants. Antioxidant mechanisms of carotenoids are divided into the four categories; sunscreening, singlet oxygen quenching, releasing excessive light energy through the xanthophyll cycle, and radical scavenging. Lutein and zeaxanthin have extremely low IC_50_ values (50% inhibitory concentration) against O_2_^−^•, OH•, and lipid peroxide compared with that of typical antioxidants, such as ascorbic acid and Trolox (Table 1) [35]. Hydrophobicity of carotenoids is extremely high, thus their IC_50_ values for scavenging against hydrophobic ROS such as DPPH radical are, in general, lower than those against hydrophilic ROS, such as ABTS radicals. On the contrary, hydrophilic antioxidants, such as ascorbic acid and Trolox, show the opposite tendency. It is meaningless to compare scavenging activity superficially, because the levels of radical scavenging activity are easily varied by experimental conditions, such as hydrophobicity and hydrophilicity of oxidants and antioxidants, atom species on which radical electrons are located, and steric bulkiness around radicals. However, the strong radical scavenging activity of carotenoid species is doubtless.

**Table 1:** IC_50_ values of some carotenoids, ascorbic acid, and Trolox against various ROS species and organic radicals.

Radical species	IC_50_ [M]			
	Lutein	Zeaxanthin	Ascorbic acid	Trolox
O_2_^-^•	37	98	>16,700	−
OH•	3.1	3.5	9,900	−
DPPH•	61	17.6	79.5	52
ABTS•	>176	53	6.8	2.6
Inhibition of lipid peroxidation	3.9	3.2	2,800	−

IC_50_ values are quoted from reference 30.

Carotenoids can also inhibit photosensitized oxidation, so that they can act as an efficient quencher of singlet oxygen. The principal quenching mechanism is electron exchange energy transfer, so-called Dexter-type energy transfer. Electron exchange between singlet oxygen and carotenoids (singlet state) forms oxygen (triplet state) and excited carotenoids (triplet state). The excited carotenoids release the energy as heat by skeletal vibration. For the efficient energy release, at least nine π-conjugated trans-alkene groups are necessary in the hydrocarbon chain. Singlet oxygen quenching efficiency of carotenoid depends on the π-conjugation length in the polyene structure. Canthaxanthin, which has additional conjugated carbonyl groups at both ends, has a second-order quenching reaction constant at 1.2 × 10^9^ M^−1^•sec^−1^ in CDCl_3_/CD_3_OD = 2/1. On the contrary, β-carotene has a much smaller reaction constant of 0.049 × 10^9^ M^−1^ sec^−1^ [36]. Quenching by chemical reaction, leading to the decomposition of carotenoids, can occur, but it is only a very minor side reaction.

## 3. Sunscreen Pigments

### 3.1. Mycosporine-Like Amino Acid (MAA)

#### 3.1.1. Structural Characteristics of MAAs

Mycosporine-like amino acids (MAAs) are one of the pigment molecules produced in cyanobacteria and algae. MAAs can also be, uncommonly, found in animals, but it is believed that these MAAs are derived from parasitic microorganisms or microorganisms taken in through ingestion, except for specific cases including metazoans [37]. The MAAs consist of an aminocyclohexenone or aminocyclohexene imine structure as the basic chromophore structure of MAA, on which various kinds of amino acids are substituted [38]. Exact stereostructure of MAAs, including amino acids substituents, is not completely clarified except for palythine and palythene. Being different from other UV absorbing antioxidants in cyanobacteria (chlorophylls, carotenoids, and scytonemin), MAAs are highly hydrophilic due to their zwitter ionic form derived from the amino acid substitution, mentioned later. The hydrophilicity further increases due to modification by sulfonic acids [39] and sugar molecules [40].

#### 3.1.2. Biosynthesis of MAAs

**Figure 1:**
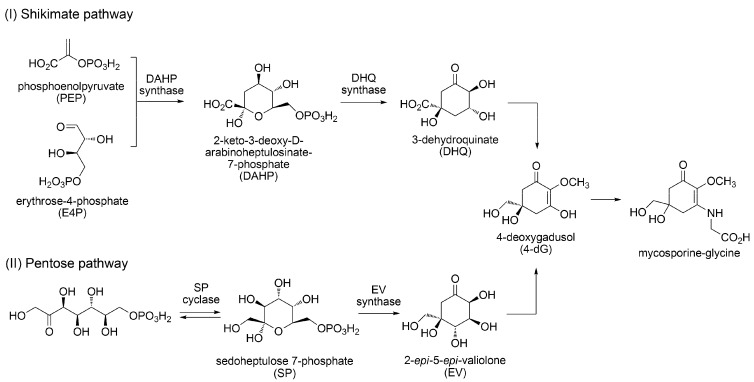
Two possible pathways for the biosynthesis of mycosporine-glycine quoted from references 41, 42 and 43.

The biosynthetic pathway of MAAs has not been completely elucidated; however, a model pathway has been proposed from studies of algae and cyanobacteria. In this pathway, it is believed that 4-deoxygadusol (4-dG) is a common intermediate, synthesized through the shikimate and pentose pathways, which is then transformed into mycosporine glycine (Figure 1) [41,42]. In *Anabaena variabilis* (ATCC 29413), the genes involved in the shikimate pathway were found as Ava_3856 (may code deoxy-D-arabino-heptulosonate phosphate synthase: DAHP synthase), Ava_3857 (coding 3-dehydoroquinate synthase: DHQ synthase), and Ava_3858 (coding O-methyl transferase). The major 4-dG synthesis pathway is believed to be shikimate pathway. On the contrary, very recently it has been found that knockout of 2-epi-5-epi-valiolone (EV) synthase, which catalyzes the different enzymatic reaction from those of DAHP synthase and DHQ synthase in the shikimate pathway, causes inhibition of shinorine synthesis in the same cyanobacterium. This result indicates a new shinorine, in other words, mycosporine-glycine synthetic pathway using sedoheptulose 7-phosphate as an original precursor [43].

**Figure 2:**
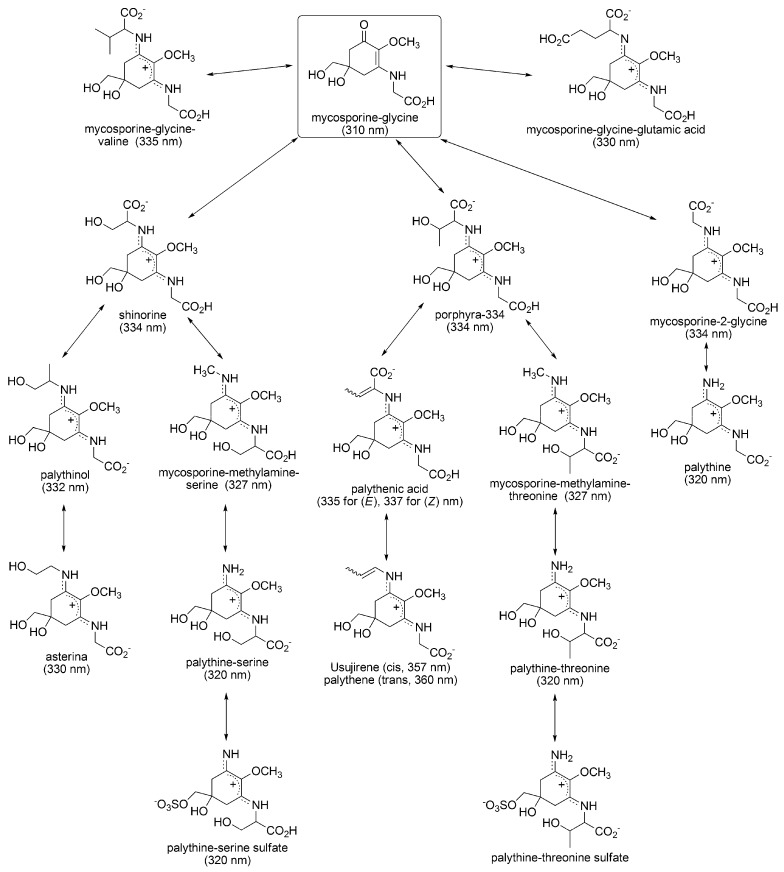
Proposed biosynthetic pathway of mycosporine-like amino acids (MAA) [38]. Absorption maximum is shown in round brackets.

Following substitution of mycosporine-glycine by various kinds of amino acids subsequently gives various MAAs (Figure 2). Substitution of valine, glutamic acid, serine, threonine, and glycine give mycosporine-glycine-valine, mycosporine-glycine-glutamic acid, shinorine, porphyra-334, and mycosporine-2-glycine, respectively. Following transformations of amino acid substituents produces various MAA derivatives. There are several reports associated with MAA biosynthesis. In *Anabaena variabilis* (ATCC 29413), the gene involved in shinorine synthesis was found as Ava_3855, which combines serine onto mycosporine-glycine. When radiolabelled glycine and serine are fed to cyanobacterium, they are incorporated into mycosporine-glycine and shinorine, respectively. Therefore, the synthetic pathway of shinorine via mycosporine-glycine is strongly supported [44]. However, other enzymes and genes involved in the biosynthesis of MAAs have not been identified. Heterologous expression in *E-coli* of the gene; NpR5600, NpR5599, and NpR5598, isolated from *Nostoc punciforme* (ATCC29133), led to the synthesis of mycosporine-glycine and expression of NpF5597 preferentially led to shinorine synthesis [45]. The synthetic pathway apparently differs from *Anabaena variabilis*. Thus, at least two putative biogenesis routes for mycosporine-glycine have been identified and other genes involved in the following MAA biosynthesis will be diverse. Sulphonation, methylation, and glycosylation of MAA are known, but the step in which these modification occur, as well as which genes are involved in these modification, are still unknown. The plausible synthetic pathways shown in Figure 2 are predicted from the structural similarities in MAAs, and details may be varied in algal species. Further research progress is expected in order to determine the biosynthetic pathway and acclimative responses in MAA synthesis, changing environmental conditions, even in diverse organisms.

#### 3.1.3. Sunscreen Properties of MAAs

The MAA biosynthetic pathway is not completely elucidated, as mentioned above; though it is well known that biosynthesis of MAAs can be promoted with UV light. MAA synthesis in cyanobacteria is enhanced by photosynthetically active radiation (PAR), UV-A (315-400 nm), and UV-B (280–315 nm) light radiation. Enhancement with UV-B light was the highest of the three light regimes [46,47]. Enhancement of MAA synthesis depends on wavelength; therefore, the involvement of specific photoreceptors to trigger a signal transduction pathway is assumed to activate biosynthesis of MAA in response to light conditions. Photoreceptors specific to UV-A and UV-B light have not been absolutely identified at present, but the possibility of indirect signal perception without specific photoreceptors was indicated in the case of UV-B irradiation [48]. UV-B light can enhance ROS production because of the higher energy of UV-B light compared to that of UV-A light. The accumulated ROS can function as signal transfer molecules, which affect antioxidative genes to turn on signal transduction for the biosynthesis of various antioxidants and antioxidant enzymes. Therefore, MAA biosynthesis can be activated in an indirect manner via ROS accumulation as a primary event caused by UV-B irradiation.

**Figure 3:**
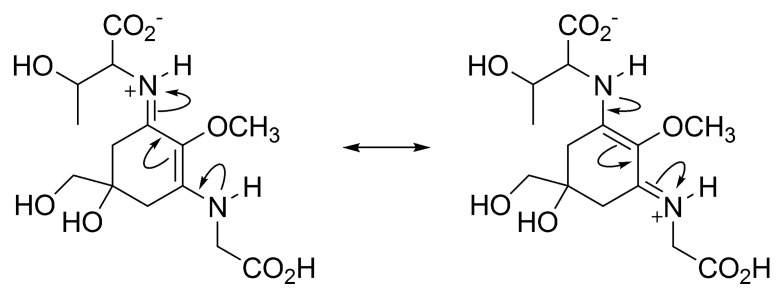
Resonance structure of protonated porphyra-334.

Many MAAs consists of an aminocyclohexene imine structure as a common chromophore. It is believed that the nitrogen atom at the imine group is likely to be protonated by an acid-base reaction with carboxylic acid in an amino acid group, to form zwitterion. The positive charge on a nitrogen atom is delocalized over the chromophore by conjugation (Figure 3). According to DFT calculations by Klisch *et al.*, it was found that the NMR chemical shift assignable to imine carbon of porphyra-334 agreed strongly with the calculation result, which was obtained using a protonated form of porphyra-334 [49]. This calculation result also supports that the configuration at the imine site is estimated as predominantly (*E*, *E*) form. The affinity for protons on imine nitrogen of lone pairs is extremely high (265.7 kcal/mol). This result indicates that MAA can function as a strong proton sponge. UV-*vis* absorption properties of MAAs are quite unique because of these structural characteristics. The absorption maxima of MAAs vary from 310 nm to 362 nm, depending on the type of amino acid substitution, and their molar extinction coefficients (ε) are extremely large (ε = 28,100–50,000 L/mol cm).

MAAs function as UV-A light absorbing agents, and can release light energy as heat without ROS production [50,51,52]. This is because the range of absorption bands of MAAs overlaps well with the UV-A range and their molar extinction coefficients are very high. Experiments on photoacoustic calorimetry revealed that more than 90% of the absorbed light energy was released into the surroundings as heat. The high energy-dissipation capacity of MAAs also contributes to their lower photodegradability. Quantum yields of photodegradation reactions of palythine and shinorine were only 1.2 × 10^−5^ and 3.4 × 10^−4^, respectively. This high stability indicates that pro-oxidant activity of degraded MAAs can almost be ignored.

#### 3.1.4. Radical Scavenging Activity of MAAs

It is known that MAAs react with several radical species, such as the 2,20-azo-bis (2-amidinopropane) dihydrochloride (AAPH) radical and the ABTS radical, which is a carbon-centered and a nitrogen-centered radical, respectively. Their scavenging activities depend on the skeletal structures of the MAAs. It was reported that ABTS radical scavenging activity of mycosporine-glycine is far greater than that of porphyra-334, shinorine, palythine, palythinol, and asterina-330, which consist of an aminocyclohexene imine structure [53,54]. Furthermore, the radical scavenging activity of MAAs change, in a pH dependent manner, and it is especially prominent in aminocyclohexene imine structures (Table 2) [54]. IC_50_ values of porphyra-334, shinorine, and asterina-330 against ABTS radicals are drastically decreased from pH 6.0 to 8.5 in a monotonic manner, though sufficient difference in the IC_50_ value of ascorbic acid is not found. On the contrary, the IC_50_ value of mycosporine-glycine decreases drastically between pH 6.0 and 7.5, but there is not a large difference between pH 7.5 and 8.5.

**Table 2:** The IC_50_ values of several MAAs against 2,2'-azinobis(3-ethylbenzothiazoline-6-sulfonic acid) (ABTS) radicals at different pH conditions.

		*p*H		
		6.0	7.5	8.5
**IC_50_ [μM]**	Mycosporine-glycine	20	4	3
	Porphyra-334	1000	400	80
	Shinorine	−	−	100
	Asterina-330	100	60	10
	Ascorbic acid	11	4	26

IC_50_ values are quoted from reference 54

**Figure 4:**
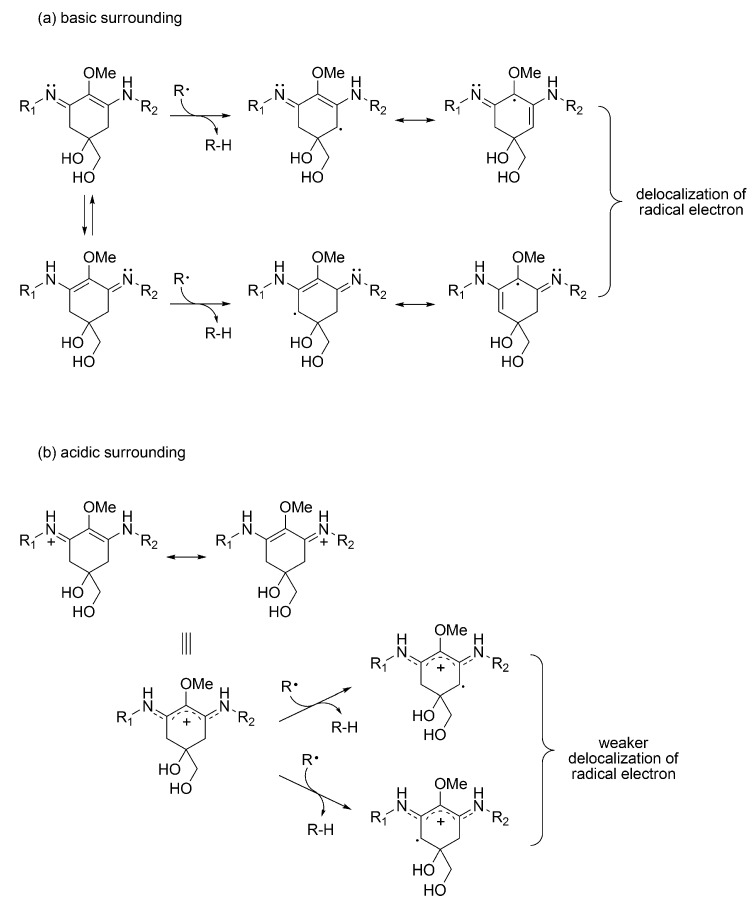
Stabilization by resonance of radical electron at the C-4 and C-6 position in iminocyclohexene-type MAA in (**a**) basic and (**b**) acidic surroundings.

The difference in reactivity likely depends on the stability of MAA-derived radical products formed by hydrogen atom abstraction (Figure 4) [55]. After abstraction of the methylene hydrogen atom at C-4 and/or C-6 position, the radical electron is delocalized by resonance over the double bonds in cyclohexene groups. This delocalization stabilizes the radical species formed. Because of the basicity, imine-type MAAs present as the protonated form as mentioned above, especially at a low pH. The radical electron in imine-type MAAs cannot be widely delocalized compared with carbonyl-type MAAs, thus imine-type MAAs decrease reactivity against radical species at a low pH. Radical scavenging activity of carbonyl-type MAAs is much greater than that of imine-type MAAs even at physiological pH conditions. Therefore, it is believed that MAAs containing an aminocyclohexenone structure play a key role in the antioxidant mechanism of MAAs. Various studies indicate that biosynthesis of MAAs is triggered by oxidative stress caused by exposure to light and heat, suggesting antioxidant function of MAAs *in vivo*. However, most MAAs discovered until now have an aminocyclohexene imine structure (imine-type MAA). During stress-induced MAA synthesis, MAAs with an aminocyclohexene imine structure may be rapidly converted to MAAs with an aminocyclohexenone structure under oxidative stress in order to enhance the antioxidant capacity *in vivo*; however such a mechanism has not been identified yet.

#### 3.1.5. MAAs on Anhydrobiosis

**Figure 5:**
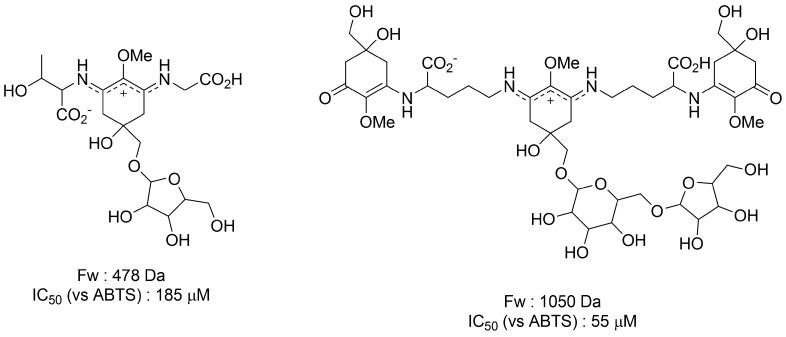
The novel glycosylated MAAs in *Nostoc commune* and their ABTS radical scavenging activities (IC_50_) [57].

Furusaki *et al.* investigated the crystal structure of palythine by X-ray crystallographic analysis and discovered that there were twelve kinds of hydrogen bonds between one palythine molecule and three water molecules [56]. This indicates that various types of hydrogen bonds are formed, not only intra- but also inter-molecularly, to form a three dimensional network. The hydrogen bond network structure with water molecules is expected to contribute to the vitrification of cells in a desiccated condition. However, this has not yet been demonstrated. Of importance is whether MAAs can really vitrify in living cells. Several glycosides of MAAs have been isolated from a terrestrial cyanobacterium *Nostoc commune* and their structures and functions have been reported. In 1995, Böhm *et al.* first discovered MAA glycosides in which galactose, glucose, xylose, gluconic acid, and glucosamine were attached by covalent bonds to imine-type and carbonyl-type MAAs [40]. Moreover, Matsui *et al.* recently discovered MAA glycosides with a pentose group [57]. They investigated ABTS radical scavenging activity of the pentose-substituted porphyra-334 and found that the IC_50_ value was 185 μM, which is almost comparable to the value obtained from Trolox (182 μM) (Figure 5). These MAA glycosides are thought to be localized in the extracellular matrix as in other MAA species. Furthermore, it is believed that they bound to extracellular polysaccharides and/or a special protein called acidic water stress protein (WspA) by physical interaction [58,59]. In *Nostoc commune*, Matsui *et al.* also discovered a novel MAA glycoside that contains two kinds of chromophores (cyclohexenone and cyclohexene imine) in one molecule (Figure 5) [57]. These chromophores have maximum absorbance at 312 nm and 340 nm, respectively. This hybrid MAA molecule can absorb a wider range of UV-A/B lights; therefore, protection from damage caused by UV-A/B irradiation is efficient. The hybrid MAA in *Nostoc commune* is the first report that indicates the possibility of MAA macromolecules via bonding between amino acids. They also revealed the IC_50_ value of this novel kind of MAA, having multi-chromophore, was 55 μM, which indicates that the radical scavenging activity of multi-chromophore type MAA is stronger than that of simple pentose-substituted MAA. MAA-attached macromolecules formed with proteins and sugars, as well as macromolecules generated by polymerization of MAAs, have a high probability for cell protection by vitrifying cells in a desiccated condition. Biosynthetic pathway and the novel function of these glycosylated MAA derivatives in cyanobacteria are still unknown. Studying the quantity and localization of these MAA-derived macromolecules, and identifying the relevant genes, will enable us to clarify the involvement in anhydrobiosis.

### 3.2. Scytonemin

#### 3.2.1. Structural Characteristics of Scytonemin

**Figure 6:**
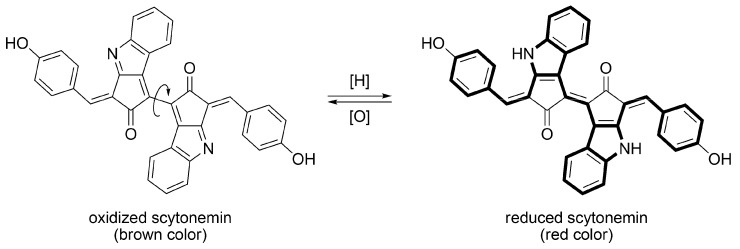
Structures of oxidized and reduced scytonemin. The π-conjugation in reduced scytonemin is drawn in a bold line.

Scytonemin is a hydrophobic pigment formed by dimerization of two polycyclic chromophores, made by the condensation of cyclopentanone and indole rings (Figure 6) [60]. In an oxidized state, the two chromophores are connected by a single bond. Therefore, they can freely rotate and prevent steric repulsion. This steric repulsion between two bulky chromophores makes a dihedral angle of about 90 degrees, so electronic interaction between them becomes very weak. On the other hand, in a reduced state, the indole ring and the benzene ring, which have 10-π and 6-π electron aromaticity, respectively, are alternately connected by double bonds. Therefore, π-conjugation is expanded by increase of planarity. Due to this structural and electrical change, the color of the compound changes from brown (oxidized state) to red (reduced state). In living cyanobacteria, scytonemin exists in the oxidized state, so that their presence is easily recognized by their brown color, observed in a bright field optical microscope. The redox state of isolated scytonemin depends on the redox condition, or acid-base condition, during the isolation process. Mixed redox forms of scytonemin are sometimes isolated. Unlike MAA species, almost no scytonemin derivatives have been discovered, except for methoxy substituted scytonemin derivatives. They are isolated from *Scytonema* sp. and their structures were determined by NMR and MS spectrometries [61].

#### 3.2.2. Biosynthesis of Scytonemin

**Figure 7:**
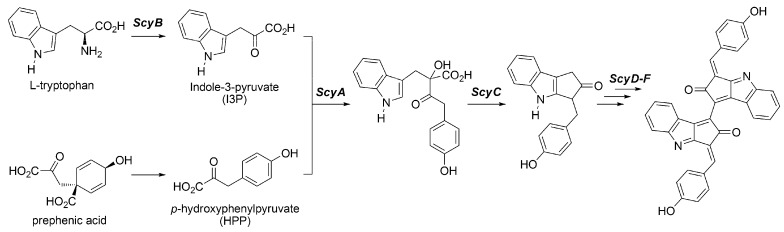
Proposed biosynthesis pathway of scytonemin.

Different from other pigments such as chlorophylls, carotenoids and MAA species, scytonemin has been found in specifically cyanobacteria. Thus, scytonemin is a unique sunscreen pigment produced only in cyanobacteria. In *Nostoc punctiforme*, the important genes involved in scytonemin biosynthesis, NpR1271, NpR1272, NpR1273, NpR1274, NpR1275, and NpR1276, have been identified and proteins coded by them are named as ScyF, E, D, C, B, and A, respectively [62]. Enzymes ScyA, B, and C are supposed to catalyze the synthesis of scytonemin monomers, while ScyD, E, and F are supposed to catalyze the dimerization of them (Figure 7). It is believed that ScyB is involved in the deamination of tryptophan to synthesize indole-3-pyruvate (I3P) because of the homology of NpR1275 with a gene involved in the synthesis of NADP-dependent oxidoreductase [63]. In addition, the amino acid sequence of ScyA has strong sequence similarity to acetolactate synthase. Therefore, ScyA is believed to catalyze an acyloin condensation from *p*-hydroxyphenylpyruvate (HPP) and I3P [64]. A characteristic of ScyC is the catalysis for the specific cyclization reaction, which has never been possible by chemical reaction. Balskus *et al.* have recently revealed its mechanism and they found that cyclization follows decarboxylation reaction [65]. Enzymes ScyD, E, and F catalyze the dimerization reaction, followed by cyclization by ScyC. However, the precise details of them are still unknown. Biosynthesized scytonemin is localized in the extracellular sheath and accumulates up to 5% of the cultured cyanobacterial colony as dry weight, although the wild-type colony sometimes accumulates much more [66].

#### 3.2.3. Sunscreen Property and Radical Scavenging Activity of Scytonemin

Biosynthesis of scytonemin is activated during UV-A/B (preferentially UV-A) light exposure in the cyanobacteria tested [33,67]. Thus, it is supposed that scytonemin functions as a photoprotective compound. It is also known that, in some anhydrobiotic cyanobacteria, repeated desiccation activates scytonemin synthesis under UV-A exposure [68,69]. The maximum absorption wavelength of scytonemin is 370 nm *in vivo*. However, it shifts towards a longer wavelength of 384 nm in a solvent after isolation. It is reported that the molar extinction coefficient of scytonemin is large (250 l/g•cm) at wavelength 384 nm [70]. It is calculated to be 136,000 l/mol•cm based on a molecular weight of 544 Da. Therefore, scytonemin is an efficient photoprotective compound due to its large extinction coefficient. The wide absorption range of scytonemin is also helpful for photoprotection, because it can cover, not only the UV-B range, but also the UV-A range. Scytonemin can protect cells by preventing 90% of the UV-A light from penetrating into the cells [71]. Matsui *et al.* studied the radical scavenging activity of scytonemin using electron spin resonance spectroscopy and discovered that the IC_50_ value of scytonemin against the ABTS radical was 36 μM [72]. Scytonemin has strong radical scavenging activity, similar to that of MAA species. These findings indicate that scytonemin is a multifunctional molecule that can absorb UV light and prevent generation of ROS, as well as scavenging ROS, even if ROS is generated by the combination of UV radiation and excessive oxidative stress.

Scytonemin is localized in the extracellular sheath occupied by proteoglycans, which is a hydrophilic environment. There is apparent contradiction because scytonemin is quite hydrophobic and barely dissolves in water. WspA is expressed at high levels in the extracellular matrix of *Nostoc commune*. It is reported that WspA synthesis is promoted by exposure to desiccation and UV light, the same as scytonemin. Scytonemin can be accumulated in the hydrophilic extracellular sheath by noncovalent bonding to the hydrophobic site in WspA [58,73]. However, it is still unknown how scytonemin and WspA complex contributes to the function of extracellular matrix in anhydrobiosis.

#### 3.2.4. Scytonemin on Anhydrobiosis

As discussed above, it is believed that scytonemin is an important molecule for the protection of cyanobacterial cells from UV irradiation. In natural environments, scytonemin shades harmful UV irradiation at the top of microbial communities. For anhydrobiosis, however, a high concentration of scytonemin is not necessarily required to recover from a desiccated state. Treatment with 100% acetone for several days, up to eight weeks, enables the extract of scytonemin at 85% purity from the extracellular sheath of desiccated cyanobacterial colonies of *Lyngbya* sp. and *Anabaena variabilis* [74]. It was found that this scytonemin-less cyanobacteria recovered almost 100% of their photosynthetic activity after rehydration, compared with the value before extraction. Scytonemin was also recovered after reculturing the rehydrated cyanobacteria. The high acetone resistance of these cyanobacteria indicates that it is possible to repeatedly produce, and extract, scytonemin from cyanobacteria without killing them. Therefore, it should be an important characteristic of scytonemin when a continuous large-scale production of scytonemin is required for industrial use.

## 4. Future Prospective

The antioxidant properties of four kinds of cyanobacterial pigments were outlined in this review. These pigments inhibit ROS production by preventing excess light energy from reaching the insides of cells by absorbing UV light. Furthermore, these pigments can suppress cell damage by directly scavenging ROS produced under environmental stress conditions such as desiccation and strong light irradiation. The multifunctional properties of these pigments are one of the important defense mechanisms in cyanobacteria to perform the oxygenic photosynthesis in various environments. Terrestrial cyanobacteria must achieve strong resistance against desiccation, and also recovery processes during rehydration to acquire anhydrobiosis characteristics. With non-reducing saccharides and specially functioning proteins, glycosylated MAA derivatives and scytonemin, localized in the extracellular matrix, may contribute, not only as antioxidants, but also as vitrification agents to prevent desiccation and rehydration damage. The bioactive substances functioning in anhydrobiosis in terrestrial cyanobacteria are particularly interesting for the industrial applications. Further research in multifunctional properties among these sunscreen pigments and other protective compounds such as saccharides, proteins, and antioxidants will progress as new analytical technology is applicable.
